# The Influence of the Second Phase on the Microstructure Evolution of the Welding Heat-Affected Zone of Q690 Steel with High Heat Input

**DOI:** 10.3390/ma17030613

**Published:** 2024-01-27

**Authors:** Huan Qi, Qihang Pang, Weijuan Li, Shouyuan Bian

**Affiliations:** 1 School of Materials and Metallurgy, University of Science and Technology Liaoning, Anshan 114051, China; henryhuanhuan@163.com; 2 Anshan Iron and Steel Group, Anshan 114006, China; 322301@ustl.edu.cn

**Keywords:** Q690 steel, second phase, high heat input, heat affected zone, microstructure

## Abstract

Q690 steel is widely used as building steel due to its excellent performance. In this paper, the microstructure evolution of the heat-affected zone of Q690 steel under simulated high heat input welding conditions was investigated. The results show that under the heat input of 150–300 kJ/cm, the microstructures of the heat-affected zone are lath bainite and granular bainite. The content of lath bainite gradually decreased with the increase in heat input, while the content of granular bainite steadily increased. The proportion of large-angle grain boundaries decreased from 51.1% to 40.3%. Overall, the average size of original austenite increased, and the precipitates changed from Ti (C, N) to Cr carbides. During the cooling process, the nucleation position of bainitic ferrite was from high to low according to the nucleation temperature, and in order of inclusions at grain boundaries, triple junctions, intragranular inclusions, bainitic ferrite/austenite phase boundaries, twin boundaries, grain boundaries, and intragranular inclusions at the bainitic ferrite/austenite phase interface. The growth rate of bainitic ferrite nucleated at the phase interface, grain boundary, and other plane defects was faster, while it was slow at the inclusions. Moreover, it was noted that the Mg-Al-Ti-O composite inclusions promote the nucleation of lath bainitic ferrite, while the Al-Ca-O inclusions do not facilitate the nucleation of bainitic ferrite.

## 1. Introduction

With the large-scale of equipment, the traditional multi-pass welding method has seriously hindered the production efficiency, and the high heat input welding technology of using >50 kJ/cm heat input to achieve single pass welding of thick plates has been widely concerned, which greatly reduces the production hours. However, the higher peak temperature and longer cooling time brought about by the increase in heat input significantly reduced the properties of the heat affected zone (HAZ) [1,2]. Therefore, improving the microstructure and properties of the coarse grain heat affected zone (CGHAZ) with the weakest performance in the HAZ has become the focus of research [3,4]. Different techniques have been explored to improve low temperature toughness of CGHAZ, in particular oxide metallurgy, which uses non-metallic oxides in steel to promote acicular ferrite (AF) nuclei and refine grains by obtaining large amounts of AF. For example, Luo et al. increased the impact energy of pearlite ferrite steel CGHAZ from 13 J to 127 J at −20 °C by adding Ti and Ti oxides to induce the nucleation of AF [5]. However, for quenched and tempered high-strength steel, the nucleation of AF is challenging due to the increase in alloy element content.

The use of high-strength steel in production can effectively reduce the weight of the structure and improve the reliability of the product [6]. Compared with traditional carbon steel, high-strength Q690 steel has higher yield strength and tensile strength, which can usually reach 690 MPa or even higher. This high strength allows Q690 steel to withstand greater loads and stresses in structural design, which is particularly important for applications where high-strength materials are required, such as long-span bridges, high-rise buildings and heavy machinery. At the same time, Q690 steel also has good toughness and weldability, and can be connected by conventional multi-pass welding and laser welding methods, which is convenient for construction and maintenance [7,8,9]. For the wide application of Q690 steel, the evolution of its CGHAZ has been studied for many years. Zhang et al. examined the influence of welding peak temperature on the heat-affected zone of Q690 steel [10]. The results showed that from 800 °C to 1150 °C, the peak temperature increases and the microstructure changes, while at the low temperature, impact toughness decreases. Chiew et al. found that HAZ is generally accompanied by several detrimental characteristics, such as large prior austenite grain size, upper bainite, martensite-austenite (M/A) constituents, and microalloy precipitates, which may lead to the lowest toughness in the heat-affected zone. Among these microstructural features, the M/A constituent (crack susceptibility) plays an important role in decreasing joint toughness [11]. The results of Hung-Wei Yen et al. show that the embrittlement in conventional S690Q steel with higher carbon content is primarily explained by the formation of lenticular martensite along prior austenite grain boundaries or packet boundaries [12]. Similarly, Li et al.’s study believed that M/A(diameter greater than 2 μm) was the main source of crack initiation [13].

However, traditional studies are usually based on very small heat input (single digits), and the welding of thick steel plates usually requires hundreds of kilojoules per centimeter of heat input, which means the evolution mechanism of HAZ of Q690 steel under the condition of high heat input welding is still immature, especially the influence of inclusions in Q690 steel on the evolution of tissues. In this paper, the microstructure evolution of Q690 steel under high heat input was studied, especially the influence of the second relative bainite ferritic core and growth.

## 2. Materials and Methods

### 2.1. Experimental Materials

**Figure 1:**
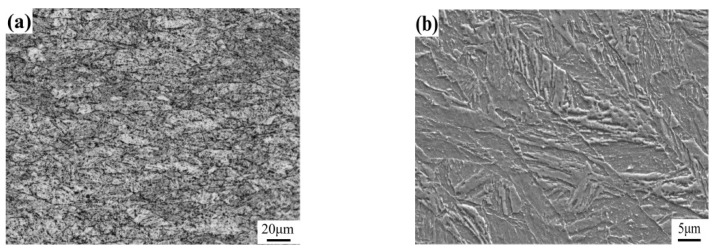
Original structure of experimental steel: (**a**) OM, (**b**) SEM.

The steel grade used in the experiment is Q690, and it was obtained directly from the steel plant in China. The base metal was hot rolled, quenched, and tempered. The thickness of the base metal is 40 mm, and its yield strength at room temperature reaches 800 MPa. According to GB/T 1591-2008 [14], the specific chemical composition and mechanical properties of Q690C used in this research are given in Table 1 and Table 2. Mechanical properties were measured three times and averaged. As shown in Figure 1, the microstructure at 1/4 of the thickness of the experimental steel is tempered martensite.

### 2.2. Simulated Welding Thermal Cycle Experiment

To study the change rule of microstructure and mechanical properties at the welding heat affected zone, the simulated welding thermal cycle experiment was conducted using a thermecmaster- 100 kN thermal simulation experimental machine. The sample was cut along the perpendicular in the rolling direction of the steel plate with dimensions of 11 × 11 × 140 mm^3^. The t_8/5_ value of different heat inputs was calculated by an empirical formula, and the welding thermal cycle curve was established. t_8/5_ represents the time from 800 °C to 500 °C during sample cooling. The calculations show the cooling times as 53 s, 71 s, 89 s, and 106 s, with corresponding cooling speeds of 5.6 °C/s, 4.2 °C/s, 3.4 °C/s, and 2.8 °C/s, and the heat inputs of 150 kJ/cm, 200 kJ/cm, 250 kJ/cm, and 300 kJ/cm, respectively. The analysis was performed at 1350 °C at a heating rate of 100 °C/s and then cooled to room temperature with different heat inputs and holding for 2 s. The t_8/5_ empirical formula is shown in Equation (1) [15]:

(1) $$t_{8/5}=\frac{E}{2\pi \lambda }\left[\frac{1}{500-t_{0}}-\frac{1}{800-t_{0}}\right]$$

where *E* is the welding heat input. *λ* is the thermal conductivity, 0.36. *t*_0_ is the initial welding temperature, 20 °C.

### 2.3. Microstructure Observation

To observe the heat-affected zone of the simulated welding thermal cycle, the sample was cut where the thermocouple was placed, polished, and then etched with 4% nitric acid alcohol solution. Subsequently, an Axio vert A1 optical microscope and an Evo MA 10 scanning electron microscope were employed to observe surface morphology, and a nano-measurer plug-in was used to measure the grain size of original austenite. An Energy Dispersive Spectrometer (EDS) was used to analyze the composition of inclusions in steel. TEM carbon extraction samples [16] were prepared according to the conventional experimental procedures, and the samples were extracted from self-made welding samples. A VL2000DX laser confocal microscope was used to observe the experimental steel in situ. In the experiment, the steel was heated to 1350 °C at a heating rate of 10 °C/s, then held for 2 s, and finally cooled to room temperature at a cooling rate of 2.8 °C/s. The laser confocal sample was cut to 1/4 of the thickness of the experimental steel. The sample diameter was 7.5 mm with a length of 2.5 mm. The samples used for EBSD analysis were intercepted on simulated welding samples, processed by mechanical vibration polishing and ion etching at room temperature, and analyzed by EBSD using a nordlysnano microscope.

## 3. Experimental Results and Analysis

### 3.1. Microstructure of Welding Heat Affected Zone

**Figure 2:**
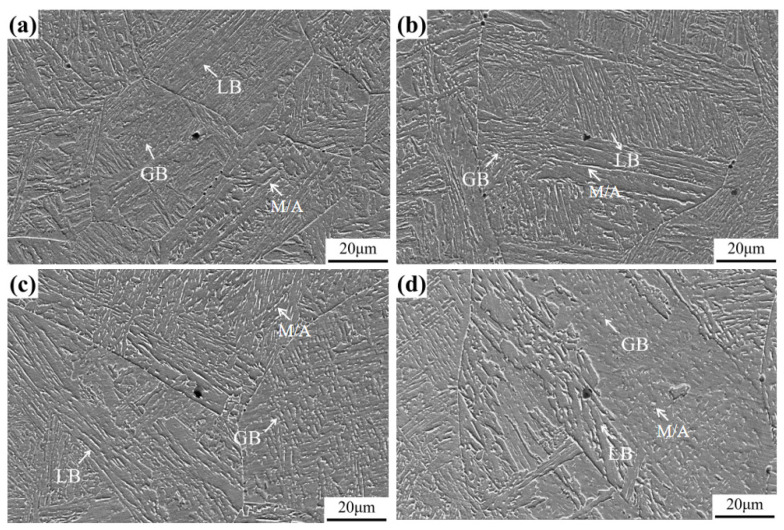
Microstructure of simulated welding heat affected zone: (**a**) Heat input—150 kJ/cm, (**b**) Heat input—200 kJ/cm, (**c**) Heat input—250 kJ/cm, (**d**) Heat input—300 kJ/cm.

The microstructure of the simulated welding heat affected zone under the heat inputs of 150–300 kJ/cm is shown in Figure 2. From Figure 2, It can be observed that the microstructure under different heat input conditions is lath bainite and granular bainite. The bainitic ferrite in lath bainite is in the form of a plate, and the M/A islands are distributed between laths in the form of long strips. The bainitic ferrite in the granular bainite is irregularly massive, and the M/A islands are also irregularly distributed on the ferrite matrix. There are many bainite blocks with different orientations in the same original austenite grain, which can be composed of lath bainite or granular bainite. With the increase in heat input, lath bainite decreases, and the granular bainite increases because the formation temperature of granular bainite is marginally higher. Due to the increase in heat input, the residence time of austenite in the high-temperature stage is prolonged, and granular bainite is developed at higher temperatures [17], leading to the inconspicuous lath characteristics.

**Figure 3:**
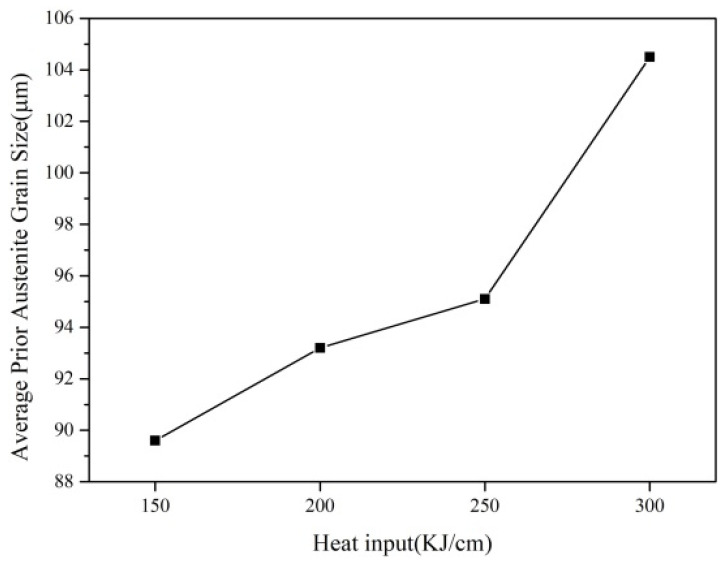
Original austenite grain size of simulated weld heat affected zone.

Figure 3 shows the statistical results of the original austenite grain size under different heat input conditions. With the increase in heat input, the original austenite grain size increased from 89.6 μm to 104.5 μm. When the heat input is greater than 250 kJ/cm, the original austenite grain size grows appreciably. This is because when the heat input is high, the cooling rate at the high-temperature stage is low, and the second phase particles that prevent the growth of austenite grains may aggregate and grow or dissolve in austenite, thus losing the function of inhibiting the grain growth, hence the austenite grains grow rapidly.

**Figure 4:**
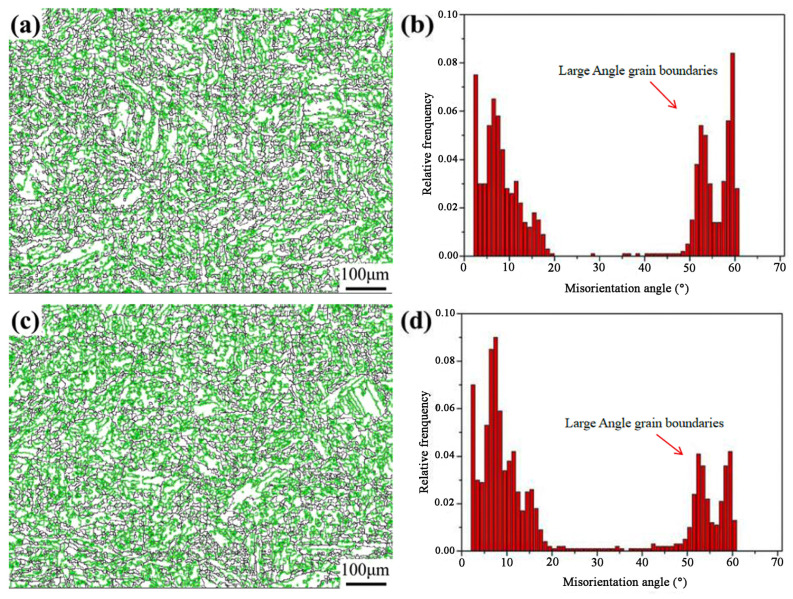
Grain boundary orientation distribution in simulated welding heat affected zone: (**a**,**b**) heat input—150 kJ/cm; (**c**,**d**) heat input—300 kJ/cm.

The grain boundary orientation distribution of samples with the heat input of 150 kJ/cm and 300 kJ/cm is shown in Figure 4. Orientation difference greater than 15° is defined as a large angle grain boundary, and 2°–15° is defined as the low angle grain boundary [17]. In the figure, the black line is a high-angle grain boundary, and the green line stands for a small-angle grain boundary. The proportion of large angle grain boundaries in the 150 kJ/cm sample is 51.1%, and that in the 300 kJ/cm sample is 40.3%. That means the heat input increases from 150 kJ/cm to 300 kJ/cm and the proportion of high-angle grain boundaries decreases accordingly. This is because the original austenite grain coarsens with the increase in heat input; the content of lath bainite decreases and the content of granular bainite increases. The original austenite grain boundary is a high-angle grain boundary, and the adjacent bainite blocks in austenite are oriented at high angles [18]. Therefore, with an increase in the heat input, the proportion of high-angle grain boundaries decreases. It must be noted that the toughness of materials is associated with the number of high-angle grain boundaries. In crack propagation, when the crack touches the high angle grain boundary, it changes its propagation path and consumes more energy, hence the toughness is improved.

### 3.2. Second Phase in Welding Heat Affected Zone

**Figure 5:**
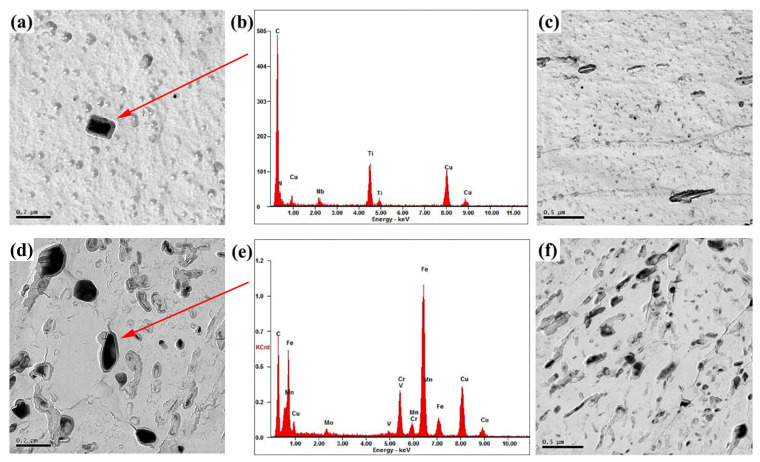
TEM observation of precipitates in welding heat affected zone: (**a**–**c**) heat input—150 kJ/cm; (**d**–**f**) heat input 300 kJ/cm.

Figure 5 shows a TEM image of precipitates at the simulated weld heat-affected zone under different heat input conditions. When the heat input is 150 kJ/cm, the Ti precipitates (C, N) exist with a diameter in the range of 15–65 nm and an average diameter of 29 nm (Figure 5a–c). When the heat input is 300 kJ/cm, the precipitates are Cr carbides with a diameter in the range of 16–140 nm and an average diameter of 62 nm (Figure 5d–f). The results show that the second phase coarsens and its average size increases with the increase in heat input; simultaneously, the phase composition also changes. When the heat input is 150 kJ/cm, the second phase particles do not grow substantially, and the average size is small due to the fast-cooling rate in the high-temperature stages, and its phase composition is Ti (C, N). Research shows that TiN dissolves at 1300–1350 °C, and a large lag effect is revealed during the dissolution of TiN. At a slower cooling rate, it continuously dissolves TiN when cooled below 1300 °C [16]. The carbide dissolution temperature of Cr_7_C_3_ is about 1890 °C, and the precipitation morphology depends on its nucleation and growth behavior at different cooling rates [19]. At 300 kJ/cm heat input, Ti (C, N) particles easily dissolve due to the slow cooling rate in the high-temperature stage, hence no Ti (C, N) particles are observed in TEM, and the second phase shows the Cr carbide. Simultaneously, the fine Cr carbides grow up at elevated temperatures, which increases their average size.

With the increase in heat input, the average size of the second phase in the heat-affected zone keeps increasing, the inhibition effect on the growth of austenite grain decreases, and the continuous coarsening of the austenite grain is accelerated. This effect is consistent with the experimental results, as shown in Figure 2 and Figure 3.

### 3.3. CLSM In Situ Observation

**Figure 6:**
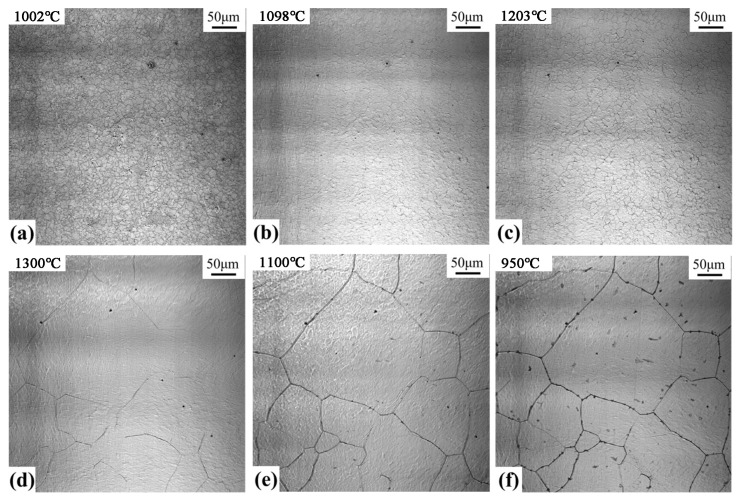
Dissolution and precipitation of inclusions during heating and cooling at 300 kJ/cm heat input: (**a**) Temperature rise—1002 °C, (**b**) Temperature rise—1098 °C, (**c**) Temperature rise—1203 °C, (**d**) Temperature rise—1300 °C, (**e**) Temperature drop—1100 °C; (**f**) Temperature drop: −950 °C.

The dissolution and precipitation of inclusions during the heating and cooling process of the welding heat affected zone with 300 kJ/cm heat input were observed with a laser confocal microscope, as shown in Figure 6. When heated to 1002 °C, numerous fine inclusions are distributed on the austenite matrix (as shown in Figure 6a). When the temperature rises to 1098 °C, several fine inclusions dissolve and disappear, and some larger inclusions are refined (Figure 6b). By increasing the temperature to 1203 °C, the small-sized inclusions further dissolve, and only a few large-sized inclusions are observed on the substrate (Figure 6c). When the temperature continues rising to 1300 °C, the fine inclusions keep on dissolving, and the existing larger inclusions are coarsened (Figure 6d). This can be related to the Ostwald ripening process of inclusions during heating [20]. Small particles dissolve and large particles grow; the driving force of Ostwald ripening explains that the interface energy between the inclusion particles and the matrix keeps decreasing. During the cooling, when the temperature reduces to 1137 °C, the inclusions precipitated in the austenite grain and on the grain boundaries are observed. By cooling to 950 °C, the number of inclusions precipitated in austenite further increases. The final distribution is shown in Figure 6f.

**Figure 7:**
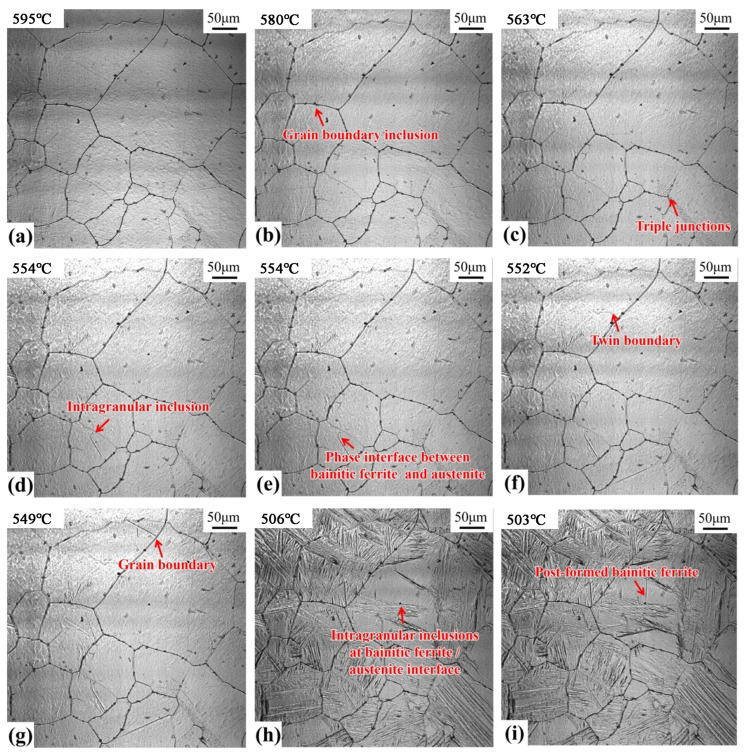
In situ observation of bainite ferrite formation in the weld heat affected zone at 300 kJ/cm heat input: (**a**) 595 °C; (**b**) 580 °C; (**c**) 563 °C; (**d**) 554 °C; (**e**) 554 °C; (**f**) 552 °C; (**g**) 549 °C; (**h**) 506 °C; (**i**) 503 °C.

Figure 7 shows the nucleation process of the bainitic ferrite in the welding heat-affected zone when 300 kJ/cm heat input is observed by a laser confocal microscope. In the image, the nucleation of bainitic ferrite is shown by the formation of surface bumps. Figure 7a is the image of the sample when the temperature decreases to 595 °C. By decreasing the temperature to 580 °C (Figure 7b), the bainite ferrite nucleation is observed at the inclusions on the austenite grain boundary. Similarly, when the temperature drops to 563 °C (Figure 7c), bainite ferrite nucleates are observed at the triple grain boundary of austenite; when the temperature drops to 554 °C (Figure 7d), strip-shaped nuclei of the bainite ferrite plate are observed on the intragranular inclusions; at the same time, bainitic ferrite rapidly forms at the phase interface between the bainitic ferrite lath formed earlier and austenite (Figure 7e). When the temperature drops further to 552 °C (Figure 7f), bainite ferrite nucleates at the twin boundary of austenite; and when the temperature drops to 549 °C (Figure 7g), bainite ferrite nucleates again at the austenite grain boundary. At 506 °C (Figure 7h), the bainitic ferrite lath formed in the austenite crystal collided with the inclusions; when the temperature marginally decreased to 503 °C (Figure 7i), the inclusion (as the nucleation core) promoted the nucleation of bainitic ferrite.

From the experimental results, it can be analyzed that bainite ferrite nucleation occurs at 7 positions in the austenite structure during the cooling process. According to the nucleation temperature (from high to low), inclusions exist at grain boundaries, trigeminal grain boundaries, intragranular inclusions, phase interfaces of bainitic ferrite/austenite, twin boundaries, and grain boundaries. The intragranular inclusions exist at bainitic ferrite/austenite phase interfaces. It can be concluded that (1) Inclusions promote the nucleation of bainite and ferrite. However, due to the various positions of inclusions at the grain boundary or in the crystal, the degree of nucleation is different because of the nucleation temperature. Normally, bainite nucleation occurs preferentially in the grain boundary because of many factors, including the high lattice distortion at the grain boundaries, the high energy, the low diffusion activation energy, the rapid migration of carbon atoms, and the formation of carbon-rich and carbon-poor regions. When the temperature of the carbon-poor region is lower than the transformation temperature of bainite ferrite, bainite ferrite begins to nucleate [21]. When the inclusions are at the grain boundary, they promote non-spontaneous nucleation [22] and the nucleation of bainitic ferrite. Therefore, the inclusions at the grain boundary become the most preferential position for the nucleation of bainitic ferrite. In addition, the inclusions in the crystal may promote the nucleation of bainitic ferrite to different degrees due to the different composition or the size of the inclusions (Figure 7d,i). The intragranular inclusions shown in Figure 7d promote the nucleation of bainitic ferrite, making its nucleation temperature higher. The intragranular inclusion shown in Figure 7i significantly promotes the nucleation of bainitic ferrite, but after collision with bainitic ferrite during its nucleation and growth, the inclusion performs as a nucleation core and promotes the nucleation of bainitic ferrite. This may be related to the further increase in strain energy around inclusions and the promotion of bainite ferrite nucleation by internal stress [23]; (2) The phase interface, grain boundary, and twin boundary promote the nucleation of bainite and ferrite. However, according to the experimental results shown in Figure 7, the priority of bainitic ferrite nucleation is in the order of trigeminal grain boundary, bainitic ferrite/austenite phase interface, twin boundary, and grain boundary. Compared with the ordinary grain boundaries, the trigeminal grain boundaries produce more distortion and higher interfacial energy. The phase boundary is a type of surface defect. Since there is a significant difference in composition and structure between the two sides of the interface, the interface energy is higher than the usual grain boundary energy. The interface energy of the twin boundary is usually low [24], and the interface energy of non-coherent Luan crystal is higher than that of coherent Luan crystal. Xu et al. explained that the priority order of the nucleation sites of bainite is the phase boundary, grain boundary, and intracrystal [25,26]. It can be inferred that the size of the phase interface, grain boundary, and twin boundary promoting the nucleation of bainite is related to the interface structure and energy. The larger interface energy produces a stronger effect on promoting nucleation and the bainite nucleates at higher temperatures.

The experimental results show that bainite nucleates and grows continuously during the cooling process, resulting in an increasing volume fraction of bainite. Bainitic ferrite formed successively at different nucleation positions shows different crystal orientations and high-angle grain boundaries, which assist in improving toughness [18]. If the nucleation position of bainite is increased by changing chemical composition and process control, more bainite blocks with different crystal orientations are expected in one austenite grain, and the mechanical properties of materials can be improved.

**Figure 8:**
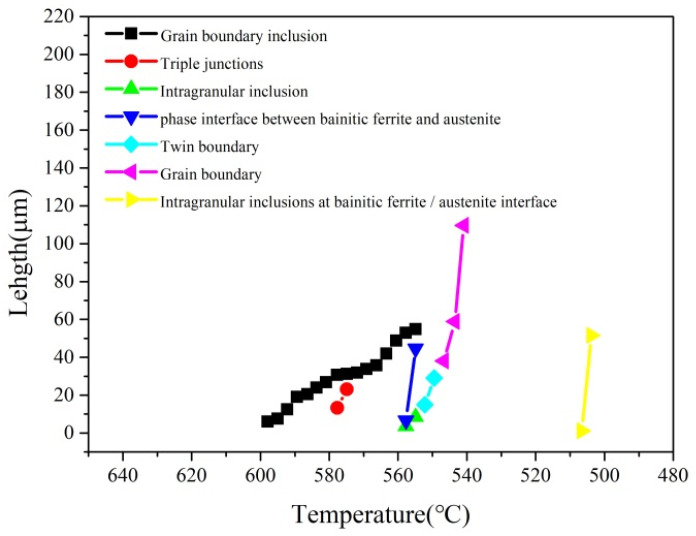
Relationship between bainite ferrite length and temperature change at each preferential nucleation position.

The kinetics of bainite formation depend on the nucleation and growth rate of bainite. Figure 8 shows the statistics of the growth rate of bainite nucleated at grain boundary inclusions, trigeminal grain boundaries, intragranular inclusions, bainite or austenite phase interface, twin boundary, grain boundary, and intragranular inclusions at bainite ferrite/austenite phase interface, which are 3.0 μm/s, 9.8 μm/s, 4.9 μm/s, 34 μm/s, 14.8 μm/s, 33.9 μm/s, and 50.4 μm/s. According to the statistical results, the growth rate of bainite from fast to slow is in the order of intragranular inclusions at the bainite ferrite/austenite phase interface, bainite/austenite phase interface, grain boundary, twin boundary, trigeminal grain boundary, intragranular inclusions, and grain boundary inclusions. This shows that the growth rate of bainite ferrite nucleated at the phase interface, grain boundary and other surface defects is fast, while the growth rate of bainite ferrite nucleated at the inclusions is slow.

According to the shear theory of bainite transformation [27], the growth of bainite is related to the diffusion of carbon atoms in ferrite and austenite. The larger diffusion coefficient of carbon atoms produces a faster growth of the bainite. Both crystal defects and temperature affect the diffusion of carbon atoms. The phase boundary, grain boundary, and other surface defects accelerate the diffusion of carbon atoms, and the bainitic ferrite nucleation is conducive to the diffusion of carbon atoms in ferrite and austenite, thus promoting the growth of bainitic ferrite. In addition, according to the experimental results, the nucleation temperature of bainitic ferrite at the surface defects (such as phase boundaries and grain boundaries) is lower than the inclusions. As the temperature decreases, the diffusion coefficient of carbon atoms decreases, and the growth rate of bainite drops. This shows that the growth rate of bainitic ferrite is affected by the phase boundary, grain boundary, other surface defects, and nucleation temperature, but also depends on the phase boundary, grain boundary, and other surface defects. The growth rate of bainite ferrite nucleated at the inclusion is slow, which promotes the refinement of bainite structure and enhances strength and toughness.

## 4. Influence of Inclusion Characteristics on Microstructure

**Figure 9:**
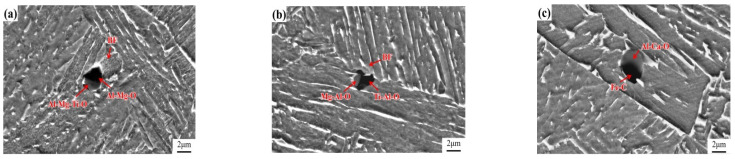
Microstructure and inclusions of welding heat affected zone: (**a**) heat input—150 kJ/cm; (**b**) heat input—200 kJ/cm; (**c**) heat input—250 kJ/cm.

Figure 9 shows the microstructure of the weld heat-affected zone under different heat inputs and the distribution of inclusions. The microstructure is composed of lath bainite and granular bainite. When the heat input is 150 kJ/cm (Figure 9a), lath bainite ferrite grows on the interface of composite inclusions. The main component of the black part in the composite inclusions is Al-Mg-O, and its maximum size is about 1.67 μm; the main component of the gray part is Al-Mg-Ti-O, and its maximum size is almost 1.45 μm; overall, the maximum size of inclusions is nearly 2.74 μm. Earlier published results showed that mg Al oxides are favorable for AF nucleation [28], and the acicular ferrite is considered a bainitic ferrite lath with a slightly higher formation temperature. The Mg-Al oxide also promotes the nucleation of lath bainite ferrite. When the heat input is 200 kJ/cm (Figure 9b), complex inclusions exist in the microstructure. The main component of the black part of the inclusions is Ti-Al-O, and its maximum size is around 1.35 μm; the main component of the gray part is Mg-Al-O, its maximum size is about 2.48 μm; and the overall maximum size of inclusions is about 2.18 μm. This composite inclusion does not directly promote the nucleation of bainitic ferrite but induces the formation of lath bainitic ferrite at the bainitic ferrite/austenite phase interface after collision with the bainitic ferrite. This observation is consistent with the results observed in Figure 7i. When the heat input is 250 kJ/cm (Figure 9c), a composite inclusion exists in the lath bainite structure. The main chemical composition of the black part of the inclusion is Fe-C, and its maximum size is abouthe 74 μm. The main chemical composition of the gray part is Al-Ca-O, and its maximum size is around 2.69 μm; the overall maximum size of inclusions is about 3.69 μm.

The mechanisms by which the inclusions promote the nucleation of bainitic ferrite include the minimum mismatch mechanism, strain induction mechanism, cation vacancy mechanism, and Mn poor region mechanism [29]. In a certain temperature range, the promotion of bainite ferrite nucleation by inclusions is related to its chemical composition and size [30]. The mismatch between Mg-O and α-Fe is 3.9%, and the mismatch between Ti-O and α-Fe is 3.1%. Studies have shown that a mismatch of less than 6% effectively promotes the nucleation of bainite ferrite [31]. The composite inclusion shown in Figure 9b contains Mg-O and Ti-O, but it does not directly induce the nucleation of bainitic ferrite. Instead, the strain-inducing mechanism and the low mismatch mechanism work together and promote the nucleation of bainitic ferrite. The mismatch estimated between Al_2_O_3_, and α-Fe is 15.2%, and the mismatch between CaO and α-Fe is noted as 18.6%. The published studies [32] also show that Al-Ca-O inclusions do not promote the bainite ferritin core. This is also confirmed by the results shown in Figure 9c.

## 5. Conclusions

1. The microstructure of the HAZ of Q690 steel under the heat inputs of 150–300 kJ/cm is composed of lath bainite and granular bainite. With the increase in heat input, lath bainite decreases, granular bainite increases, and the proportion of large angle grain boundary decreases from 51.1% to 40.3%. Overall, the original austenite grain size increased from 89.6 μm to 104.5 μm.
2. When the heat input is 150 kJ/cm, the precipitates in the heat affected zone are Ti (C, N), with an average diameter of 29 nm; When the heat input is 300 kJ/cm, the precipitates are Cr carbides with an average diameter of 62 nm.
3. During the cooling process of austenite in the heat-affected zone, the nucleation positions of bainite ferrite from high to low exist according to the nucleation temperature, inclusions at the grain boundary, trigeminal grain boundary, intragranular inclusions, bainite ferrite/austenite phase interface, twin boundary, and grain boundary. The intragranular inclusions exist at the bainite ferrite/austenite phase interface.
4. The growth rate of the bainite ferrite nucleated at the phase interface, grain boundary, and other surface defects is faster, while the bainite ferrite nucleated at the inclusions is slower.
5. Mg-Al-Ti-O composite inclusions promote the nucleation of lath bainite ferrite, but Al-Ca-O inclusions do not facilitate this.

## Figures and Tables

**Table 1:** Chemical composition of experimental steel (%).

C	Si	Mn	P	S	Al	Nb	Cr	Ni	Cu	Ca	Ti	Mg	N	O
0.07	0.22	1.57	0.01	0.0024	0.03	0.01	0.24	0.46	0.32	0.0026	0.01	0.002	0.0036	0.002

**Table 2:** Mechanical properties of experimental steel.

State	Yield Strength (MPa)	Tensile Strength (MPa)	Elongation (%)	Impact Toughness at −20 °C (J)
Quench + temper	800	836	20.5	295

## Data Availability

All the raw data supporting the conclusion of this paper were provided by the authors.
